# Genetic Diversity Linked to Haplotype Variation in the World Core Collection of *Trifolium subterraneum* for Boron Toxicity Tolerance Provides Valuable Markers for Pasture Breeding

**DOI:** 10.3389/fpls.2019.01043

**Published:** 2019-08-30

**Authors:** Hediyeh Tahghighi, William Erskine, Richard G. Bennett, Philipp E. Bayer, Maria Pazos-Navarro, Parwinder Kaur

**Affiliations:** ^1^Centre for Plant Genetics and Breeding, School of Agriculture and Environment, The University of Western Australia, Perth, WA, Australia; ^2^Institute of Agriculture, The University of Western Australia, Perth, WA, Australia; ^3^School of Biological Sciences, The University of Western Australia, Perth, WA, Australia

**Keywords:** abiotic stress, boron toxicity, genome-wide association study, haplotype analyses, hydroponic, forage legumes, subterranean clover

## Abstract

In alkaline soils in arid and semi-arid areas toxic concentrations of the micronutrient boron (B) are problematic for many cereal and legume crops. Molecular markers have been developed for B toxicity in cereals and *Medicago*. There is a need for such tools in clovers—*Trifolium*. To this end, we undertook a genome-wide association study (GWAS) with a diversity panel of subterranean clover (*Trifolium subterraneum* L.), an established model pasture legume for genetic and genomic analyses for the genus. The panel comprised 124 *T. subterraneum* genotypes (97 core collection accessions and 27 Australian cultivars). Substantial and useful diversity in B toxicity tolerance was found in *T. subterraneum*. Such variation was continuously distributed and exhibited a high broad sense heritability *H* *^2^* = 0.92. Among the subspecies of *T. subterraneum*, ssp. *brachycalycinum* was most susceptible to B toxicity (*P* < 0.05). From the GWAS, the most important discoveries were single-nucleotide polymorphisms (SNPs) located on Chr 1, 2, and 3, which mapped to haplotype blocks providing potential genes for a B toxicity tolerance assay and meriting further investigation. A SNP identified on Chr 1 aligned with *Medicago truncatula* respiratory burst oxidase-like protein (TSub_ g2235). This protein is known to respond to abiotic and biotic stimuli. The identification of these novel potential genes and their use to design markers for marker-assisted selection offer a pathway in pasture legumes to manage B toxicity by exploiting B tolerance.

## Introduction

Boron (B) is one of the essential micronutrients for healthy plant growth (Tomić et al., 2015) and is available to plants as boric acid (Reid, 2014). Due to its small molecular size and high membrane permeability in comparison to many other nutrients, the uptake and diffusion of B can be difficult for plants to control (Reid, 2014). Boron deficiency and toxicity are known to have adverse effects on agricultural production around the world (Oertli and Kohl, 1961; Nable et al., 1997; Hamilton et al., 2015). Although B deficiency is relatively easy to manage using B-rich fertilizers, B toxicity is more difficult to manage. Soil B concentration can be reduced by leaching, and B availability can be modified by pH adjustment, but this is impractical on a large scale (Yau and Ryan, 2008). Therefore, the use of genetic variation and plant breeding for tolerance is likely the best way to overcome toxicity (Yau and Ryan, 2008).

Boron toxicity mostly occurs in dry areas with an alkaline soil pH, particularly above pH 9, and in areas with low rainfall or in heavier clay soils, where B does not readily leach into deep soil layers below the root zone (Yau and Ryan, 2008). In 1983, a widespread B toxicity problem was reported in South Australia (Cartwright et al., 1984)—recently estimated to affect ∼4.9 million hectares (31%) of the agricultural zone in South Australia (Howie, 2012), and ∼15% in Western Australia (Lacey and Davies, 2009) have been identified as at risk of B toxicity. A concentration of B in the range of only 10 to 54 mg kg^-1^ in the soil inhibits plant growth (Javid et al., 2015), and soils in the southern Australian cropping region can reach 52 mg kg^-1^ B (Nuttall et al., 2003). Yield losses due to B toxicity have been reported for cereals (Cartwright et al., 1984; Paull et al., 1988), annual medics (*Medicago* spp.), field pea (*Pisum sativum* L.) (Paull et al., 1992), and lentil (*Lens culinaris* Medik.) (Yau and Erskine, 2000).

Strategies to cope with low and high soil B vary among plant species and genotypes (Reid, 2014). Plants cells are able to adjust the flow of most nutrients by selective membrane transport proteins, but in this regard, B is exceptional as it exists as uncharged boric acid at physiological pH and is therefore highly permeable through the lipid bilayers that form the basis of biological membranes (Reid, 2014). The three known pathways by which B enters and exits cells are: 1) passive, bidirectional diffusion through the lipid bilayer; 2) passive bidirectional diffusion through selective or non-selective channels; and 3) active efflux pumping (Reid, 2014). Hayes and Reid (2004) showed that a B-tolerant barley cultivar (Sahara) was able to maintain an internal B concentration lower than the external medium, presumably with an associated need for energy to preserve the gradient across the plasma membrane.

Boron toxicity symptoms vary with its mobility within the plant (de Abreu Neto et al., 2017). In common crop species, B is largely immobile once inside the cell wall, which leads to an accumulation at the leaf margins where the xylem vessels terminate (Reid, 2014), thereby causing chlorosis or necrosis of leaf tips and margins in older leaves (Brown and Shelp, 1997; Yau and Ryan, 2008). Lentil, barley (*Hordeum vulgare* L.), alfalfa (*Medicago sativa* L.), faba bean (*Vicia faba* L.), chickpea (*Cicer arietinum* L.), bread wheat (*Triticum aestivum* L.), durum wheat (*T. durum* Desf.), vetch (*Vicia* spp.), and field pea exhibit this type of B toxicity symptom (Yau and Ryan, 2008).

Reid et al. (2004) demonstrated that B inhibited growth by 40 to 60% in a monocot (barley), dicot (*Arabidopsis*—*Arabidopsis thaliana* L.) and an alga (*Chara*) when its soluble concentration reached 10 mM in the growth medium. Additionally, Kohl and Oertli (1961) reported the same concentration of B caused necrotic leaf margins in various plants. There is clearly variation in B tolerance among plant species and even among cultivars of the same species (Nable, 1988), indicating that natural variation in B toxicity tolerance exists within species, which could be used for selection and breeding of tolerant genotypes (Reid et al., 2004), leading to the development of several screening methodologies in crop species (Paull et al., 1992; Yau and Ryan, 2008; Schnurbusch et al., 2010; Javid et al., 2015; Bennett et al., 2017).

*Arabidopsis* has been used as a model of B tolerance to identify genes involved in B uptake and translocation—AtBOR1 and AtNIP5;1 (Takano et al., 2002; Takano et al., 2006), which conferred tolerance to plants under B deficient conditions. AtBOR4 and OxAtTIP5;1 are over-expression of an AtBOR1 paralog and AtTIP5;1, respectively, in transgenic *Arabidopsis* and encode transport molecules that prevent or regulate excess intercellular B (Miwa et al., 2007; Pang et al., 2010). Other studies have identified homologous genes related to B toxicity tolerance in other species: HvBot1 in barley (Sutton et al., 2007), MtNIP3 in the model legume *Medicago truncatula* (Bogacki et al., 2013), along with a single chromosomal region controlling tolerance to B in lentil (Kaur et al., 2014), two additive loci with incomplete dominance that admitted excess B tolerance in peas (Bagheri et al., 1996), and the Bo1 marker allele in durum and bread wheat (Schnurbusch et al., 2007; Schnurbusch et al., 2008). However, there are no reports of the genetic basis of B tolerance in *Trifolium*.

Subterranean clover (*Trifolium subterraneum* L.) is the most important sown annual pasture legume species in southern Australia and is grown over an estimated area of 29 million ha in the 250 to 1200 mm annual average rainfall band (Nichols et al., 2013). *T. subterraneum* is established as a model for *Trifolium* for genetic and genomic studies (Kaur et al., 2017a) on the basis of its diploidy (2n = 16), self-pollinating habit, and presence of major genomic resources. The species consists of three subspecies: 1) ssp. *subterraneum*, 2) ssp. *yanninicum*, and 3) ssp. *brachycalycinum*, of which *subterraneum* and *yanninicum* are adapted to acidic soils, and ssp. *brachycalycinum* is better adapted to neutral–alkaline soils where B is often problematic (Nichols et al., 2013). Although there is no information available on variation in B toxicity tolerance within the genus *Trifolium*, *T. subterraneum* has a wide natural distribution, which includes various soil types, presumably with variable B content, leading to the expectation that there may be a wide range of tolerance to B toxicity in this species.

The objectives of this study, using *T. subterraneum* as a *Trifolium* model, were to 1) develop a hydroponic screening system and identify a suitable concentration of B to differentiate B tolerance, 2) investigate variation for B toxicity tolerance in a wide range of germ plasm of *T. subterraneum*, and 3) investigate the genetic and molecular basis for B toxicity tolerance in *T. subterraneum* by using the candidate gene approach. We tested the specific hypotheses that: 1) there exists significant level of variation for boron toxicity tolerance within the existing *T. subterraneum* germ plasm diversity panel, 2) ssp. *brachycalycinum* is most likely to demonstrate tolerance to excess B among the three subspecies, and 3) a genome-wide association study (GWAS) will indicate potential genomic associations with B tolerance in *T. subterraneum*. The outcome of this study will help to enhance the efficiency of breeding for B toxicity tolerance in *T. subterraneum* and other *Trifolium* species. Increasing the tolerance of *T. subterraneum* to B toxicity may have a direct productivity benefit in soils with high B levels in the subsoil enabling plants to better access subsoil moisture reserves in dry seasons (Holloway and Alston, 1992).

## Materials and Methods

### Plant material: A Diversity Panel of Core Collection Lines and Cultivars

A diverse panel of 124 *T. subterraneum* genotypes (**Supplementary Table S1**) was selected for the study, which included 97 core collection accessions (Nichols et al., 2013) and 27 diverse Australian cultivars (Kaur et al., 2017b). The core collection was developed by K. Ghamkhar, R. Appels and R. Snowball to represent the genetic diversity within the world collection of >10,000 phenotypes (Nichols et al., 2013; Ghamkhar et al., 2015). Selection of the core collection followed the methodology of Ghamkhar et al. (2008) to identify a subset of 760 lines, on the basis of 1) diversity for eco-geographical data from their sites of collection; and 2) agro-morphological data obtained by the Australian Trifolium Genetic Resource Centre (ATGRC) of the Department of Agriculture and Food Western Australia (DAFWA). DNA was then extracted from leaf material of each short-listed line, and 48 single-sequence repeat (SSR) primers, spread across each of the eight *T. subterraneum* chromosomes, were selected from the results of Ghamkhar et al. (2012) to identify the most diverse lines. Analysis using MSTRAT software (Gouesnard et al., 2001) to optimize maximum diversity within the minimum number of lines, resulted in an optimum core collection of 97 lines, covering 80.1% of the genetic diversity within the whole *T. subterraneum* collection. For these wild accessions with passport data, a total of 19 bioclimatic variables representing the climate of collection sites were derived from the WorldClim database (Hijmans et al., 2005).

### Protocol Development for B Toxicity Phenotyping

All phenotyping experiments were carried out in the Plant Growth Facility at The University of Western Australia. A preliminary experiment was conducted to develop a hydroponic screening system for B toxicity tolerance in *T. subterraneum* and also to identify the level of B which showed the maximum discrimination among a selection of genotypes. Ten diverse genotypes of *T. subterraneum* (**Supplementary Table S1**) were subjected to four concentrations of B (0, 15, 30, 45 mg B L^-1^) in a hydroponic system under controlled temperature and photoperiod, an adaptation of the method reported in Bennett et al. (2017). The temperature was set at 24/20^°^C day/night and a 20-h photoperiod supplied by LED lights (4:3 ratio of model 108D18-V12 tubes from S-Tech Lighting, Australia and AP67L series tubes from Valoya, Helsinki, Finland). Forty seeds of each genotype were scarified to ensure uniform germination, and seeds were placed in plastic Petri dishes on moist filter paper to imbibe. Petri dishes were wrapped in Parafilm to avoid evaporation and aluminum foil to maintain seeds in darkness. The Petri dishes were stored at 15^°^C for 2 days. Then, for each treatment, 10 seeds of each genotype were sown into moist peat plugs within Styrofoam trays (Garden city plastic, PLT288S). One Styrofoam tray was allocated to each B treatment and placed in separate storage tubs (35L Icon Plastics) in the controlled environment room. The experimental design was based on a strip-plot with full replication among treatments. Genotypes were arranged in rows, and rows were randomized in each B treatment. Seedlings were watered immediately after sowing and again after 24 h with tap water. After a further 24 h (Day 5), 20 L of tap water was added to each storage tub to float the Styrofoam trays. The final number of individuals was greater than 5 for all genotype–treatment combinations. On day 8, the water in the tubs was replaced with a nutrient solution in DI water (adapted from Hoagland and Arnon, 1938) (**Supplementary Table S2**). The pH was maintained between 6 and 7 with bi-weekly adjustments using KOH to raise pH and H_3_PO_4_ to lower pH. On day 14, B (as H_3_BO_3_) was added into the hydroponic solution to achieve the desired concentration for treatments. On day 19, individual plants were scored for severity of B toxicity leaf symptoms using a 0.0- to 8.0-rating scale adapted for *T. subterraneum* from that used by Bagheri et al. (1992) (**Supplementary Table S3**). Leaf symptom scores were chosen as the B toxicity metric as they are routinely used in phenotyping similar legume species for B toxicity (Yau and Ryan, 2008) Leaf symptom scores for each B treatment were analyzed separately using “LSD.test” (agricolae package) in RStudio (Version 0.99.484, RStudio, Inc. R Core Team 2009-2015). The B treatment that provided the greatest level of discrimination (15 mg B L^-1^) was selected for further screening of the panel of 125 genotypes.

### Diversity Panel Screening for B Toxicity

Screening the panel of 125 genotypes (**Supplementary Table S1**) was conducted in two sub-experiments, with genotypes allocated randomly to sub-experiments. Each sub-experiment contained four hydroponic culture tubs subjected to identical growth conditions, making a total of eight “blocks” for the 125 genotypes’ screening. To test and correct for block effects, each tub contained five “check” cultivars (Antas, Dalkeith, Gosse, Izmir, and Losa) arranged randomly as partial replicates and 15 “test” entries in rows of up to ten individual plants (**Supplementary Figure S1**). Test entries were randomly allocated to tubs. Seeds were germinated, planted, and grown for 14 days as previously described. Five days after the plants were transferred to Hoagland’s solution, we observed some mild leaf symptoms on some individuals that could potentially confound B toxicity symptom expression. Hence, all plants were scored for leaf damage prior to the addition of B, and these data were used as a covariate for correction in the final analysis as described below. On day 14, 15 mg B L^-1^ (as H_3_BO_3_) was added to the hydroponic solution. Boron exposure was increased from 5 to 7 days when screening the 125 genotypes to improve the phenotyping result in this wider selection of germ plasm. Plants were scored individually for B toxicity symptoms on day 21 (**Supplementary Figures S2**, **S3A** and **Supplementary Table S3**). The final number of individuals was greater than 3 for all genotype–treatment combinations. After correction for block effects and prior leaf damage, the corrected means of B toxicity score (**Supplementary Figure S3B**) were used for further analysis.

A covariate was applied to each tub to correct for block effects by averaging the B toxicity scores of all five check lines in each tub. This covariate and a covariate derived from prior leaf damage scores were used to correct the average B toxicity score of each of the 125 genotypes (on an individual plant basis) using “UNIANOVA” in SPSS (IBM Corp., 2013). This corrected mean B toxicity score (B score) was plotted and used in the GWAS, and in further analysis of correlations between B tolerance and data from passport information. The B score was also compared to the climate at the collection site of genotypes using Bioclimatic variables (Hijmans et al., 2005; Nichols et al., 2013) (**Supplementary Table S1**) as described below.

Differences in the B score among non-continuous variables in the passport data (Soil texture, country of origin, subspecies and category) were analyzed by one-way ANOVA in RStudio with B score as the dependent variable and results were tabulated and plotted (Box plot RStudio default function). Continuous variables (latitude, longitude, altitude, soil pH and 19 BioClim variables) were analyzed by ANOVA in RStudio to produce Rcorr correlation coefficients (Hmisc package) and their significance (*R* ^2^ and *P* value).

### Genotyping of the Diversity Panel and Genome-Wide Associations

Phenotypic B tolerance information obtained from the core collection of 97 accessions and the 27 elite Australian cultivars of *T. subterraneum* were associated with specific regions in the advanced assembly (Tsub_Refv2.0) (Kaur et al., 2017a) using GWAS analyses. Genomic DNA (gDNA) was extracted from a single plant of each of the 124 genotypes of *T. subterraneum* and sequenced. High-quality whole-genome resequencing (WGRS) data were generated for all 124 of these accessions and cultivars as described by Kaur et al. (2017b). SNP identification was conducted using samtools and bcftools (Li et al., 2009; Li, 2011), then SNPs with at least one heterozygous allele, those with an Minor Allele Frequency (MAF) ≤ 5%, and those that were not present in at least one individual were removed to keep only homozygous SNPs and remove errors of mis-mapping heterozygotes. This lead to removal of clustered SNPs, which is further confounded by the relatively low population. QQ plot was conducted to evaluate the effect of low population size (**Supplementary Figure S4**). Consecutive SNPs were merged using PLINK v1.9 (Purcell et al., 2007; Chang et al., 2015) into haplotype blocks if their *r* ^2^ values were above 0.8. Linkage disequilibrium was visualized using Haploview v4.2 (Barrett et al., 2005).

The population structure for GWAS was of two sub-populations: the first sub-population comprised 27 cultivars released in Southern Australia for grazing; while the second sub-population of 97 accessions was a core germ plasm collection—a stratified sample of the world collection of *T. subterraneum* (Kaur et al., 2017b). Despite these two sub-populations, a principal component analysis reported in Kaur et al. (2017b) revealed the diversity between the sub-species: ssp. *subterraneum*, ssp. *yanninicum* and ssp. *brachycalycinum*. Four principal components were used to correct for population stratification. Individuals are split up in three subpopulations corresponding to the three sub-species (**Supplementary Figure S5**). GWAS was conducted *via* a logistic regression using the four principal components as covariates to correct for population stratification using PLINK v1.90b3.42 (Chang et al., 2015) (**Supplementary Figure S5**). BLASTP v2.2.30+ was used to link the identified genes with known genes within the annotations of *M. truncatula* Mt4.0v2 (Tang et al., 2014) and *Arabidopsis thaliana* (TAIR10) (Haas et al., 2005; Lamesch et al., 2012).

### Marker-Trait Association Studies and Putative Candidate Gene Analysis

Each significant marker-trait association (MTA) resulting from the GWAS was checked for any overlaps with haplotype blocks with *r* ^2^ values above 0.8. In which case, sequences 25-bp upstream and downstream from the SNP were extracted from the reference and were used to design PCR-ready markers for MAS for this B toxicity tolerance trait in primer3 v2.3.7 (Koressaar and Remm, 2007; Untergasser et al., 2012) (settings: primer product size, 250–500; primer optimum size, 300; primer minimum temperature, 55°C; optimal temperature, 57°C; maximum temperature, 60°C).

Putative candidate genes were proposed for each significant MTA by extracting the genes upstream, downstream, or overlapping with GWAS candidate SNPs.

## Results

### Phenotypic Traits and Boron Toxicity Tolerance

To establish a suitable screening system and to determine the best concentration of B for phenotypic traits in a hydroponic system, ten genotypes of *T. subterraneum* were tested under four different concentrations of B (preliminary experiment). Tip chlorosis and necrosis were apparent in leaves after 5 days of B treatment, and plants were scored (**Supplementary Figure S2**). ANOVA indicated that both treatment and line had a significant effect (P < 0.05) on the score, with a significant interaction between these factors. Overall, the severity of symptoms increased with increasing B concentration (*P* < 2.2e-16). An LSD test in RStudio for leaf symptom score revealed that 15 mg B L^-1^ provided the greatest level of discrimination among genotypes, and so this concentration was selected to screen the panel of 125 genotypes (**Table 1**).

**Table 1:** Effect of four different concentrations of boron for 10 genotypes of *T. subterraneum* in preliminary experiment.

	B = 0		B = 15		B = 30		B = 45	
Line name	Mean	Significance group	Mean	Significance group	Mean	Significance group	Mean	Significance group
L08/019450B-UNI	0.56	a	2.60	bc	3.06	cd	3.38	d
L19/070116A	0.44	a	2.86	bc	3.36	bcd	4.14	bc
L24/083931A	0.00	b	1.81	de	3.50	bc	3.56	cd
L25/083945C	0.00	b	1.50	ef	3.17	cd	4.20	bc
L28/083990D	0.33	ab	3.93	a	3.83	b	4.33	ab
L31/089762C	0.00	b	2.14	cde	2.93	d	4.75	ab
L102/Daliak	0.06	b	1.13	f	3.00	d	4.33	ab
L122/Trikkala	0.00	b	2.29	cd	4.67	a	4.93	a
L123/Woogenellup	0.00	b	3.00	b	3.50	bc	4.56	ab
L124/Yarloop	0.00	b	2.75	bc	3.75	b	4.75	ab

B, Boron (0, 15, 30, 45 mg B L^-1^).

^abcdef^Means within columns not sharing a common letter are significantly different (P < 0.05).

**Figure 1:**
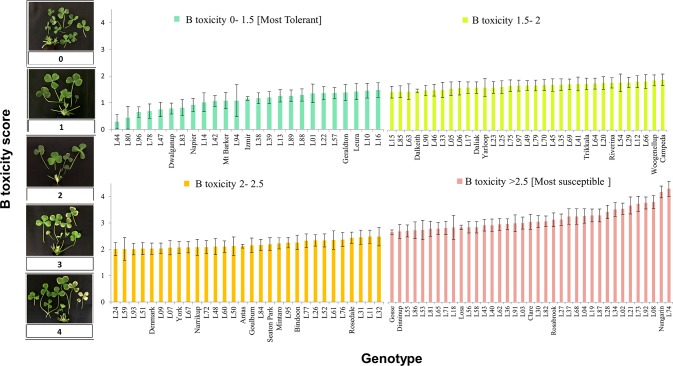
Boron toxicity symptom score for diverse germ plasm of *T. subterraneum* after correction for tub effects and the confounding prior leaf damage (on an individual basis). Cultivars are named on the x axis, and the remaining genotypes are referred to as a line name (L01 to L97) that can be cross referenced to the GRC identity using **Supplementary Table S1**.

In the subsequent screening experiment, 125 genotypes of *T. subterraneum* were subject to 15 mg B L^-1^. Among the 125 accessions, there was a continuous distribution of tolerance to B toxicity (**Figure 1**). The genotype most tolerant to excess B concentration was L44 (ssp. *subterraneum*) with an average B score of 0.3 (se 0.27). The most tolerant among the cultivars of *T. subterraneum* tested was Dwalganup, which is also a *subterraneum* ssp., with a B score of 0.8 (se 0.20) (**Figure 1** and **Supplementary Table S1**).

In terms of susceptibility, L74 (ssp. *subterraneum*) showed the most severe B toxicity symptom with an average B score of 4.1 (se 0.27). This genotype was collected from an area with clayey soil texture and pH = 7.3 (**Figure 1** and **Supplementary Table S1**). Among the cultivars, Nungarin (ssp. *subterraneum*) was the most susceptible with a B score of 3.9 (se 0.21) (**Figure 1** and **Supplementary Table S1**).

Associations between continuous variables of origin (latitude, longitude, altitude, soil pH, and 19 BioClim variables) were tested by Pearson’s correlation (**Supplementary Figure S6** and **Supplementary Table S4**). We anticipated a significant correlation between B score and soil pH. However, the strongest correlation with B score was longitude (*P* value <0.1, *R* ^2^ = 0.029). The analysis of B scores compared among discontinuous variables (soil texture, country of origin, subspecies, and category) (**Supplementary Figures S7A-D**) indicated a significant difference (*P* value <0.05) existed between B toxicity symptoms of different subspecies (**Supplementary Figure S7C**). The *post hoc* LSD test showed subspecies *brachycalycinum* was significantly more susceptible than ssp. *subterraneum* or *yanninicum* (*P*-value <0.05) (**Supplementary Table S5**). Soil pH at collections sites ranged from 6 to 9 (mean value = 7.3) at ssp. *brachycalycinum* sites and from 5 to 9 (mean value = 6.3) in ssp. *subterraneum* sites (**Supplementary Figure S8** and **Supplementary Table S1**). Soil pH data were not available for ssp. *yanninicum* accessions (**Supplementary Figure S8**). Comparing the 28 cultivars with the 97 wild accessions, the means for B toxicity tolerance of the groups were similar as were the ranges (**Supplementary Figure S7B**).

### Associating SNPs to Gene Models and PCR-Ready Markers to Track Haplotype Variation

Potential genes were proposed for each significant MTA by extracting the genes upstream, downstream or overlapping with GWAS candidate SNPs. BLASTP was used to search for homologues of proteins encoded by the candidate genes within *M. truncatula* Mt4.0v2 and *Arabidopsis thaliana* (TAIR10) database.

**Figure 2:**
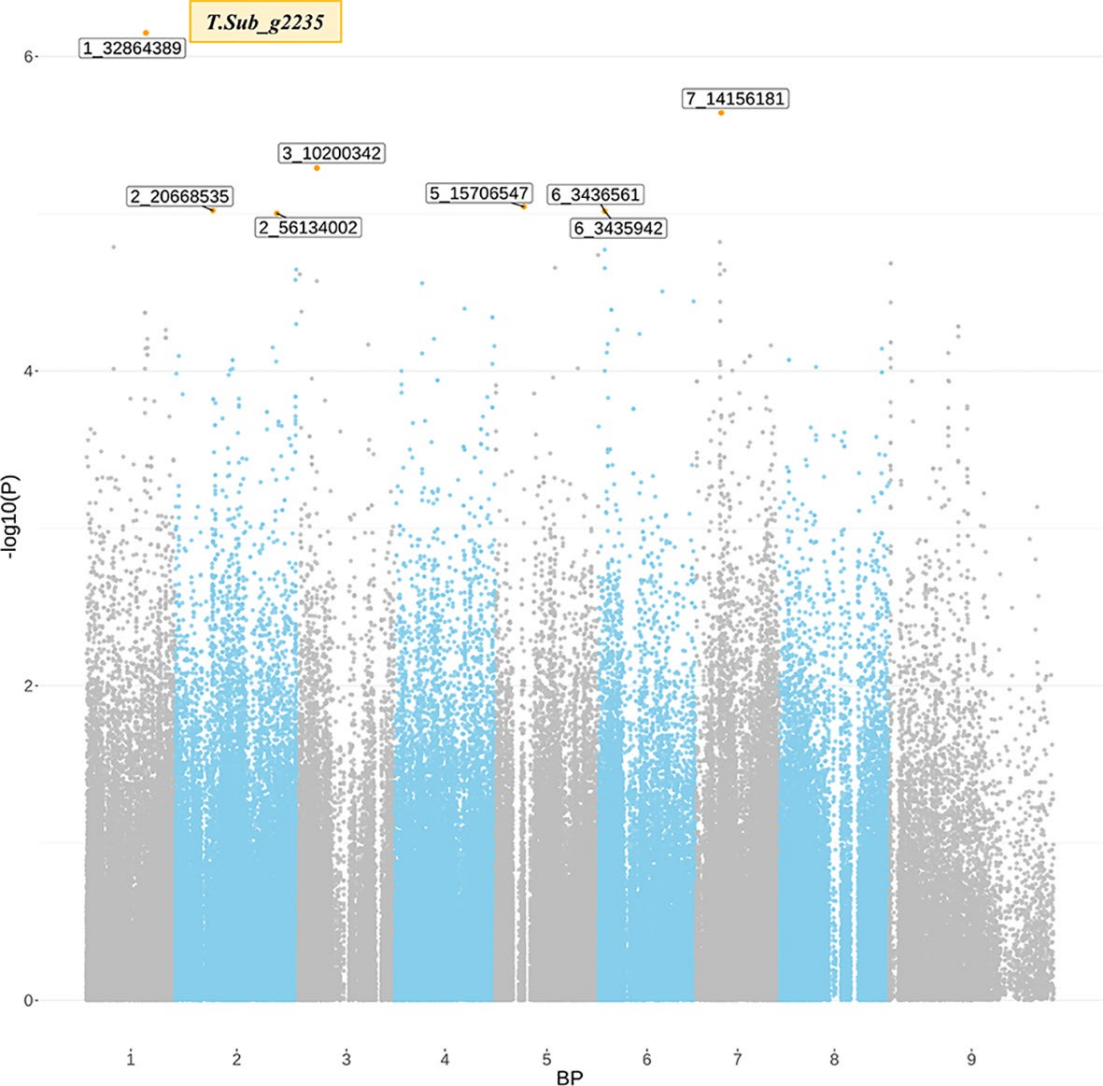
Significant marker-trait associations (MTAs) for boron toxicity tolerance detected through genome-wide association analysis using the diverse germ plasm panel of *Trifolium subterraneum*. The *x*-axis indicates the SNP location along the eight chromosomes of *T. subterraneum*, *y*-axis in each graph represents −log 10*P* for the *P* value of the MTA. The gray line marks the threshold for genome-wide significance (*P* value = −log, 10*P* > 4.0) can be considered as significantly associated.

**Figure 3:**
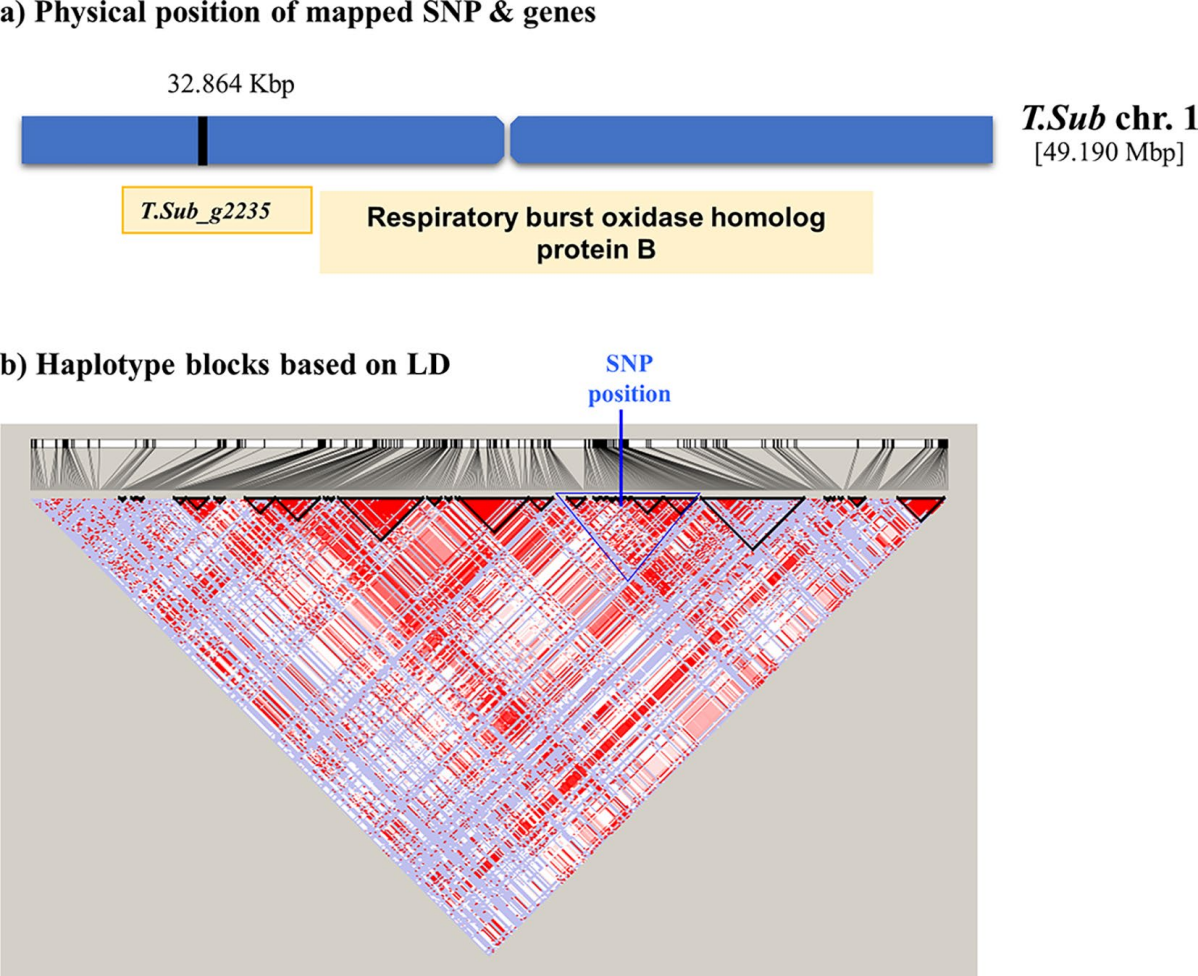
Scheme of the genomic region with the haplotype block on Chr 1 in *Trifolium subterraneum* (advanced Tsub_Rv2.0) showing **(A)** the physical position of the boron toxicity tolerance candidate gene and **(B)** haplotype blocks based on LD.

The GWAS identified eight markers which reached suggestive *P* value below 1e^-5^ on chromosomes 1, 2, 3, 5, 6, and 7 associated with the B trait (**Figure 2** ****and**** **Table 2**). QQ plot suggested a relatively weak effect due to the low population size (**Supplementary Figure S4**). The SNPs located on Chr 1, 2, and 3 were mapped in haplotype blocks containing 21, 13, and 5 other SNPs with a total length of 366.62, 240.97, and 13.43 kbp, respectively (**Figure 3** and **Table 3**). The significant SNP identified on chromosome 1 was located on the region of candidate gene TSub_ g2235 positioned between 32,860,391 and 32,866,822 with a total exon length of 2548 (**Table 2**) (Kaur et al., 2017a). Two significant SNPs were identified on Chr 2. For the first of these, an upstream endonuclease/exonuclease/phosphatase family protein (Tsub_g4776) and a downstream subtilisin-like serine protease (Tsub_g4777) were identified at a distance of 28878 and 20045 bp, respectively, from the suggestive SNP. For the second, an upstream calcium-binding EF-hand protein (Tsub_g7559) and a downstream pinoid-binding protein 1 (Tsub_g7560) were identified at a distance of 23628 and 5415 bp, respectively, from the suggestive SNP (**Table 2**).

**Table 2:** Significant genomic associations identified using the phenotyping data for the boron toxicity tolerance in *T. subterraneum*.

GWAS SNP position	CHR	UNADJ P value	BETA value	**Alleles	Overlapping gene	Upstream gene [distance from SNP]	Downstream gene [distance from SNP]	Candidate genes	Candidate Gene ID	Length	Best blast hit Arabidopsis Tair10 (“upstream,” “downstream”)	Best blast hit Medicago V4.0 (“upstream,” “downstream”)
*1_32864389	1	7.09E-07	0.547	A/G	TSub_g2235			1	TSub_g2235	848aa	AT1G09090.2	Medtr1g083290.1
*2_20668535	2	9.53E-06	–0.6432	A/G		Tsub_g4776 [-28878 bp]	Tsub_g4777 [20045 bp]	2		417aa, 440aa	“No significant hit,” “AT4G34980.1”	“Medtr1g054775.1,” “Medtr4g073540.1”
*2_56134002	2	9.95E-06	0.9289	A/G		Tsub_g7559 [-23628 bp]	Tsub_g7560 [5415 bp]	2		115aa, 115aa	“AT4G27280.1,” “AT5G54490.1”	“Medtr2g081300.1,” “Medtr2g081350.1”
*3_10200342	3	5.12E-06	0.3168	C/A		Tsub_g9589 [-2943 bp]	Tsub_g9590 [289 bp]	2		727aa, 220aa	“AT1G06840.1,” “AT1G26910.1”	“Medtr3g062590.1,” “Medtr3g062600.1”
5_15706547	5	9.01E-06	–0.6432	G/A		Tsub_g16463 [-1804 bp]	Tsub_g16464 [43166 bp]	2		237aa, 516aa	“AT1G60060.1,” “AT1G64940.1”	“Medtr5g034840.1,” “Medtr5g034900.1”
6_3435942	6	9.62E-06	–0.3637	A/G	Tsub_g19611			1	Tsub_g19611	496aa	“AT1G48900.1”	Medtr4g010050.1
6_3436561	6	9.62E-06	–0.3637	T/G	Tsub_g19611			1	Tsub_g19611	496aa	“AT1G48900.1”	Medtr4g010050.1
7_14156181	7	2.28E-06	–0.43	A/G		TSub_g22842 [-3094 bp]	Tsub_g22843 [57 bp]	2		143aa, 79aa	“AT2G18660.1,” “No significant hits for g22843”	“Medtr3g107770.2,” “No significant hit for g22843”

*Haplotype Block **Alleles: G/T,A means that G is reference, T,A are alternative alleles in the population Omitted three SNPs on chromosome 2 that behaved identical to 2_56134002 (2_56134932, 2_56138979, 2_56139128).

**Table 3:** PCR ready markers designed using primer3 for the boron toxicity tolerance significant SNP identified in the haplotype blocks.

SNP_Chromosome	SNP_position	Haplotype block start	Haplotype block end	Haplotype block length	SNPs in block	Forward Primer	Reverse Primer	Product size
1	32864389	32638043	33004659	366616	22	CCATTGGAACGGCTCATCTG	CCCTGACTGGCCTTTGACTA	176
2	20668535	20639732	20748806	109074	2	ACCTTCTCTCCAGCTGCAAT	ACCTTCTCTCCAGCTGCAAT	172
2	56134002	56107242	56140215	32973	4	TGGGATGGGTAGCTCAACAG	GTGCGATCATTGGTCACTCC	232
2	56134932	56107242	56140215	32973	4	TGGGATGGGTAGCTCAACAG	GTGCGATCATTGGTCACTCC	232
2	56138979	56107242	56140215	32973	4	TGGGATGGGTAGCTCAACAG	GTGCGATCATTGGTCACTCC	232
2	56139128	56107242	56140215	32973	4	TGGGATGGGTAGCTCAACAG	GTGCGATCATTGGTCACTCC	232
3	10200342	10199687	10213121	13434	6	TTTGTCCGGTCCCATCATCA	GTAACATCTCGCCGGTCCTA	245

An upstream leucine-rich repeat receptor-like protein kinase (Tsub_g9589) and a downstream ribosomal protein L16p/L10e family (Tsub_g9590) was identified for the significant SNP on Chr 3 at a genetic distance of 2943/289 bp, respectively (**Table 2**). On Chr 5, an upstream transcription factor-like protein (Tsub_g16463) and a downstream cytochrome P450 family protein (Tsub_g16464) were detected at the distance of 1804 and 43166 bp, respectively, from the suggestive SNP. Two significant SNPs were found on Chr 6, both being in the region of potential gene (TSub_19611) which aligned with signal recognition particle 54-kDa protein in the *M. truncatula* database (**Table 2**). On Chr 7, we identified an upstream rare lipoprotein A-like double-psi beta-barrel protein (TSub_g22842), and no significant hits for a downstream (Tsub_g22843) at the distance of 3094 and 57 bp, respectively, from the suggestive SNP (**Table 2**).

The haplotype block containing the MTA SNPs on Chr 1, 2, and 3 with total length of 366,616, 109,074, and 32,973 bp, and 13,434 bp, respectively, was used to design PCR-ready markers for MAS for this B toxicity tolerance trait (**Table 3**).

## Discussion

The present study was designed to estimate variation in B stress tolerance and to identify potential B stress-responsive genes in *Trifolium* using *T. subterraneum* as a model. The study is the first report to demonstrate that substantial useful variation in B toxicity tolerance exists in *T. subterraneum*. Furthermore, the high broad-sense heritability *H* *^2^* = 0.92 indicates that the trait is little influenced by environmental conditions. Variation in B toxicity tolerance has previously been reported for cereals—barley and wheat, and legumes—medics, peas and lentils (Nable, 1988; Paull et al., 1988; Paull et al., 1992; Bagheri et al., 1994; Yau and Erskine, 2000; Schnurbusch et al., 2008). Among *T. subterraneum* cultivars tested, Dwalganup and Nungarin were the most tolerant and susceptible to B toxicity, respectively. Clearly response to selection for B stress tolerance can be anticipated.

Significant MTAs for B toxicity tolerance were detected through GWAS analysis with the most significant discovery being the SNPs located on chromosome 1, 2, 3, which mapped into haplotype blocks. The potential gene on Chr 1 (TSub_g2235) aligned with *M. truncatula* respiratory burst oxidase-like protein and respiratory burst oxidase homolog (RBOH) protein B in *A. thaliana*. Respiratory burst NADPH oxidase is found in plant proteins, such as respiratory burst NADPH oxidase protein, which produces reactive oxygen species (ROS) as a defence mechanism (Suzuki et al., 2011). Respiratory burst oxidase homologues in plants are plasma membrane enzymes which produce ROS. They participate in a variety of mechanisms, such as cell elongation and abiotic stress signaling pathways, hormonal signaling, and pathogen response (Montiel et al., 2016; Arthikala et al., 2017). Recent studies have revealed that RBOHs participate in legume–rhizobia interaction (Montiel et al., 2016). Ozhuner et al. (2013) described B toxicity symptoms as cell wall biosynthesis degradation, inhibition of cell division, and elongation and metabolic decline by binding to the ATP, NADH, and NADPH component of the ribose. Our results suggest that RBOHs may also be involved in B toxicity tolerance in *T. subterraneum*.

BLAST search revealed that some of the other SNPs identified in MTAs have high-sequence similarities with potential genes known for plant stress responses (**Table 2**). Based on the results of the current study, these derived proteins may also be expressed in B toxicity conditions in *T. subterraneum*. Being in haplotype blocks, these genes identified on chr 1, 2, and 3 are the most stable and potential for designing molecular markers to track haplotype variation for this trait (**Table 3**). We plan to now functionally validate these genes found associated with B toxicity in subterranean clover using the CRISPR-Cas system in a follow-up study.

Although the corresponding proteins for the B transporter/channel genes AtBOR1 and AtNIP5;1 in *Arabidopsis* are responsible for B uptake in B deficient conditions (Takano et al., 2006), similar proteins in barley and *M. truncatula* have been found to be linked to B toxicity tolerance (Reid, 2007; Sutton et al., 2007; Bogacki et al., 2013). However, in the present study, no linkage was found between B toxicity tolerance and AtBOR1, AtNIP5;1 in *T. subterraneum*.

Molecular markers have been identified for selection of B toxicity tolerance in other plant species. Tolerance to excess of B is controlled by a single gene in the model legume *M. truncatula* (Bogacki et al., 2013), barley (Sutton et al., 2007), and lentil (Kaur et al., 2014). Based on our phenotypic and genotypic results, this trait could be controlled by more than one gene in *T. subterraneum*.

The hydroponic system developed herein provides an efficient, rapid (21 days) method to screen nutrient toxicity and deficiency in breeding studies. This is the first report of hydroponic screening for B toxicity tolerance in *T. subterraneum*. Hydroponics have previously been used as a rapid method for B toxicity tolerance screening in barley, *Brassica rapa* L., wheat, rice (*Oryza sativa* L.), and field pea (Jefferies et al., 1999; Kaur et al., 2006; Schnurbusch et al., 2007; Pallotta et al., 2014; de Abreu Neto et al., 2017; Bennett et al., 2017), B toxicity and salinity tolerance screening in field pea (Javid et al., 2015), and aluminum tolerance screening in barley and wheat (Baier et al., 1995; Ma et al., 1997). Screening for abiotic stress tolerance in the field is difficult due to environmental heterogeneity and variation of mineral content in soil (Stoddard et al., 2006). This research has provided a robust, high-throughput hydroponic protocol for screening B toxicity which could be readily applicable to screen other plant species and/or for other abiotic stresses.

In previous hydroponic B toxicity screening studies, the B concentrations used ranged from 162 mg L^-1^ in wheat (Schnurbusch et al., 2007) to 8 mg L^-1^ in rice (de Abreu Neto et al., 2017). In the latter study, 8 mg B L^-1^ induced severe toxicity in many varieties of rice (de Abreu Neto et al., 2017). Wheat appears to more tolerant, and rice more susceptible to B concentration compared with *T. subterraneum*.

Boron toxicity is problematic in soils with high pH (Yau and Ryan, 2008). Our expectation was that ssp. *brachycalycinum*, which is commonly found on alkaline soils, was more likely to demonstrate B tolerance than the other two subspecies of *T. subterraneum*. However, B toxicity tolerance was not significantly correlated with any passport data or BIOCLIM variables, including soil pH (**Supplementary Table S4**) and, among the three subspecies, *brachycalycinum* was the most susceptible species for B toxicity. Therefore, our expectation was not met. A possible explanation for these results is that the *brachycalycinum* genotypes tested here did not come from highly alkaline soils. B has relatively high availability in soils of pH 5 to 6.5, with availability then dropping as pH increases to 8.5. In soils above pH 8.5, B once again becomes highly available (Dwivedi et al., 1992). This reduced availability of B in neutral to moderately alkaline soils is particularly prevalent in soils with high calcium content as B has the tendency to bind with Ca in the soil (Dwivedi et al., 1992). Some studies have demonstrated that adding lime to acidic soil increased soil pH to a more moderate pH and, consequently, could result in lower concentrations of B in pea and barley plants tissue (Gupta and Macleod, 1981; Dwivedi et al., 1992). The *brachycalycinum* genotypes tested here were collected from soils with pH ranging from 6 to 9, with most (48%) collected from soil pH 6.5 to 7.5, where B would have poor availability. In contrast, the ssp. *subterraneum* genotypes tested in the current study were mostly (70%) collected in soils with pH less than 6.5. As previously highlighted, B is readily available in soil pH 5 to 6.5, consistent with our results indicating that the most tolerant genotypes were found in ssp. *subterraneum*.

In conclusion, this study demonstrated substantial variation in tolerance to B toxicity in *T. subterraneum* germ plasm, which was genetically dissected by GWAS. Potential genes were identified through GWAS associated with B toxicity tolerance that merit further investigation. The high throughput hydroponic system developed here could be applicable to other plants for screening for abiotic stress. Furthermore, tolerant cultivars, such as Dwalganup and Napier, would be priorities for use in soil types with potential for B toxicity. The results from this study provide valuable, new information for both plant breeding and gene validation studies using CRISPR technology in *T. subterraneum*.

## Author Contributions

HT performed the boron phenotyping research under the guidance of PK, WE, RB, PB, and MP-N and wrote the article with contributions from all. PK designed and performed the sequencing experiments and conducted the bioinformatics analysis with PB. All authors read the article and approved the content.

## Funding

This study was conducted by the Centre for Plant Genetics and Breeding (PGB) at The University of Western Australia (UWA). Funding for this work was also provided by an Australian Research Council Linkage Grant (LP100200085), UWA RMF Grant, Meat and Livestock Australia (MLA grant B.PBE.037) and the Department of Agriculture and Food Western Australia (DAFWA).

## Conflict of Interest Statement

The authors declare that the research was conducted in the absence of any commercial or financial relationships that could be construed as a potential conflict of interest.
