# Supplementary figures and images for: Genetic Diversity Linked to Haplotype Variation in the World Core Collection of Trifolium subterraneum for Boron Toxicity Tolerance Provides Valuable Markers for Pasture Breeding
Source: Front Plant Sci. 2019 Aug 30;10:1043. doi: 10.3389/fpls.2019.01043 (PMC6729137; doi:10.3389/fpls.2019.01043)

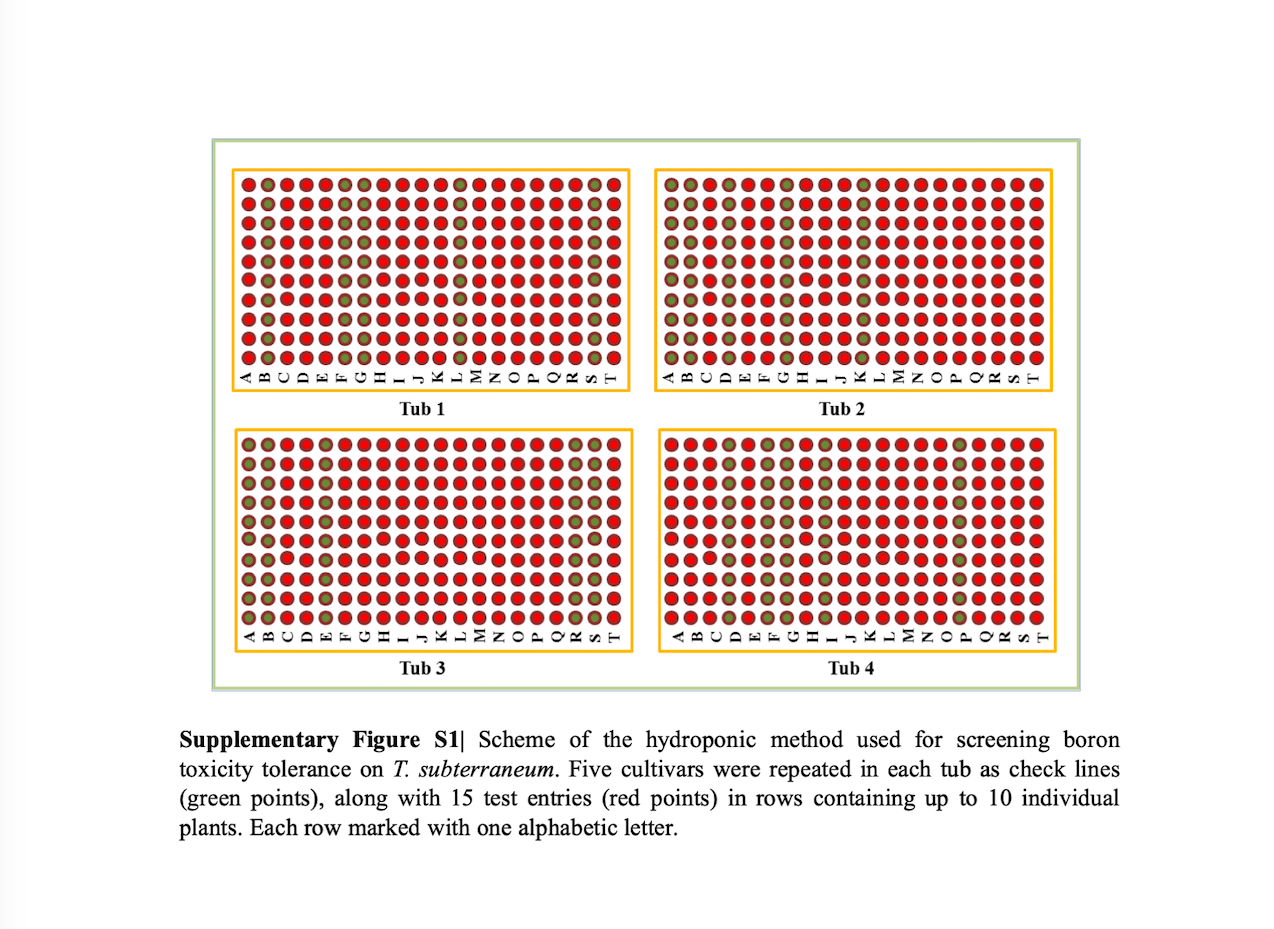

Supplement: Supplementary file 1 [file Image_1.png]

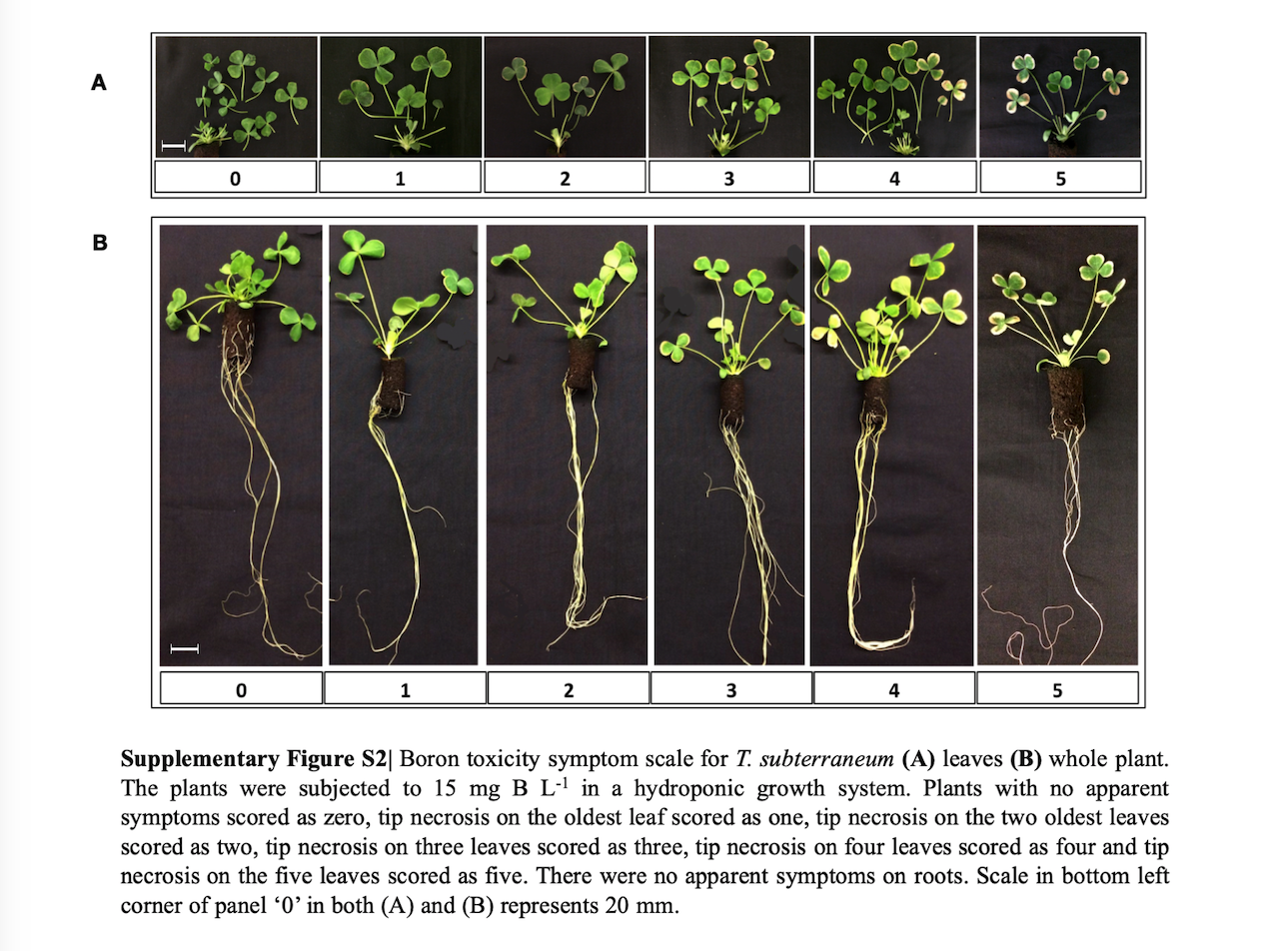

Supplement: Supplementary file 2 [file Image_2.png]

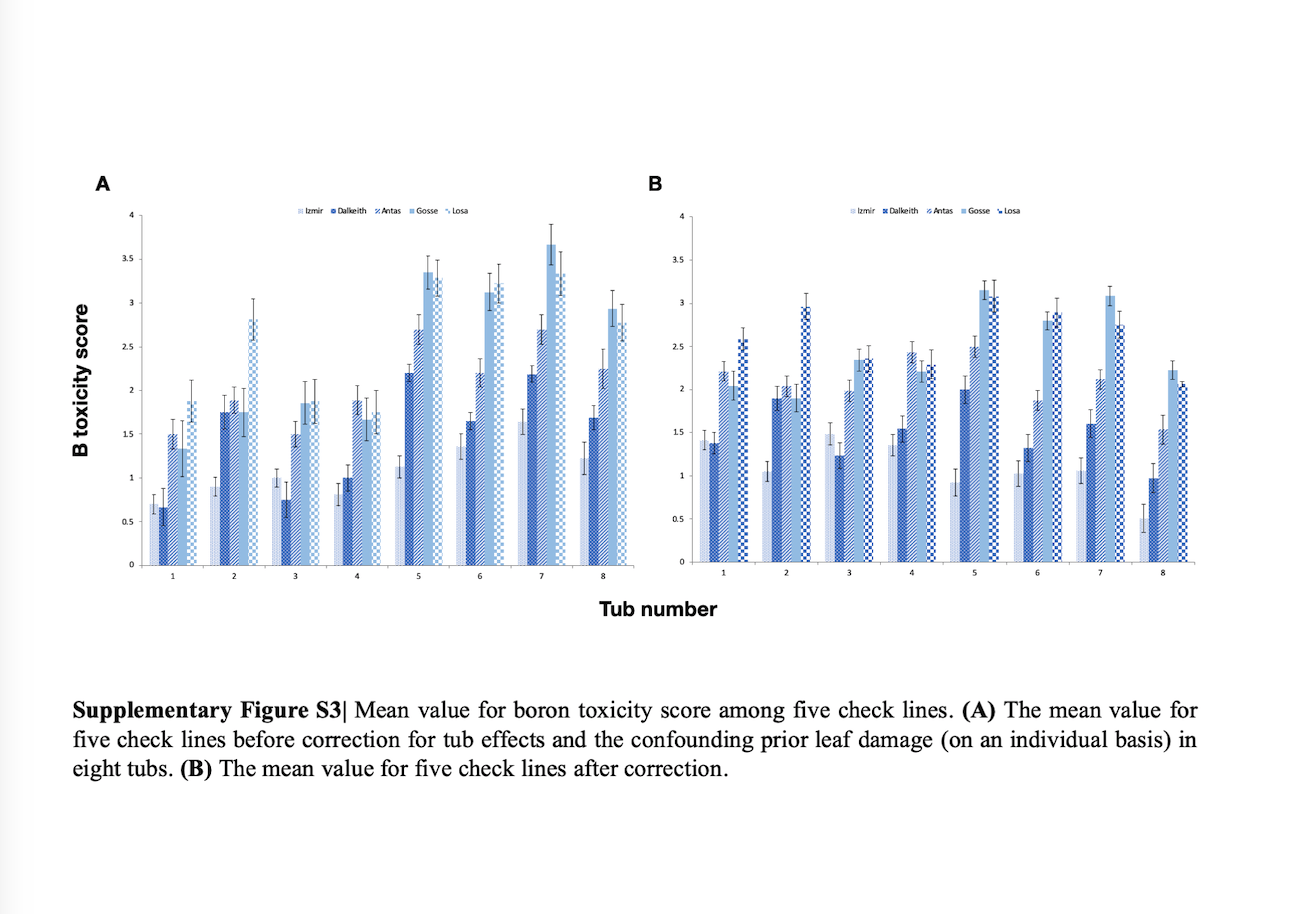

Supplement: Supplementary file 3 [file Image_3.png]

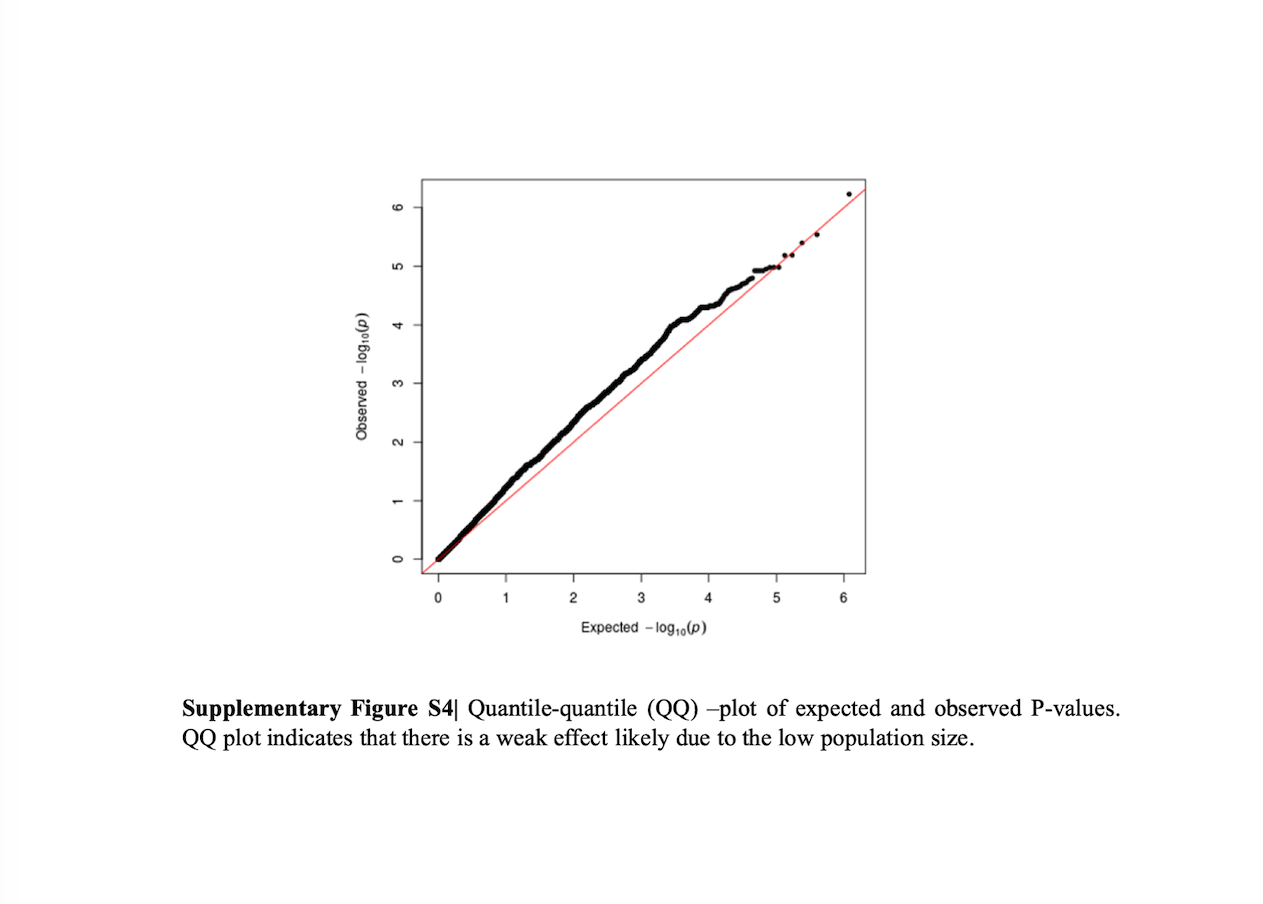

Supplement: Supplementary file 4 [file Image_4.png]

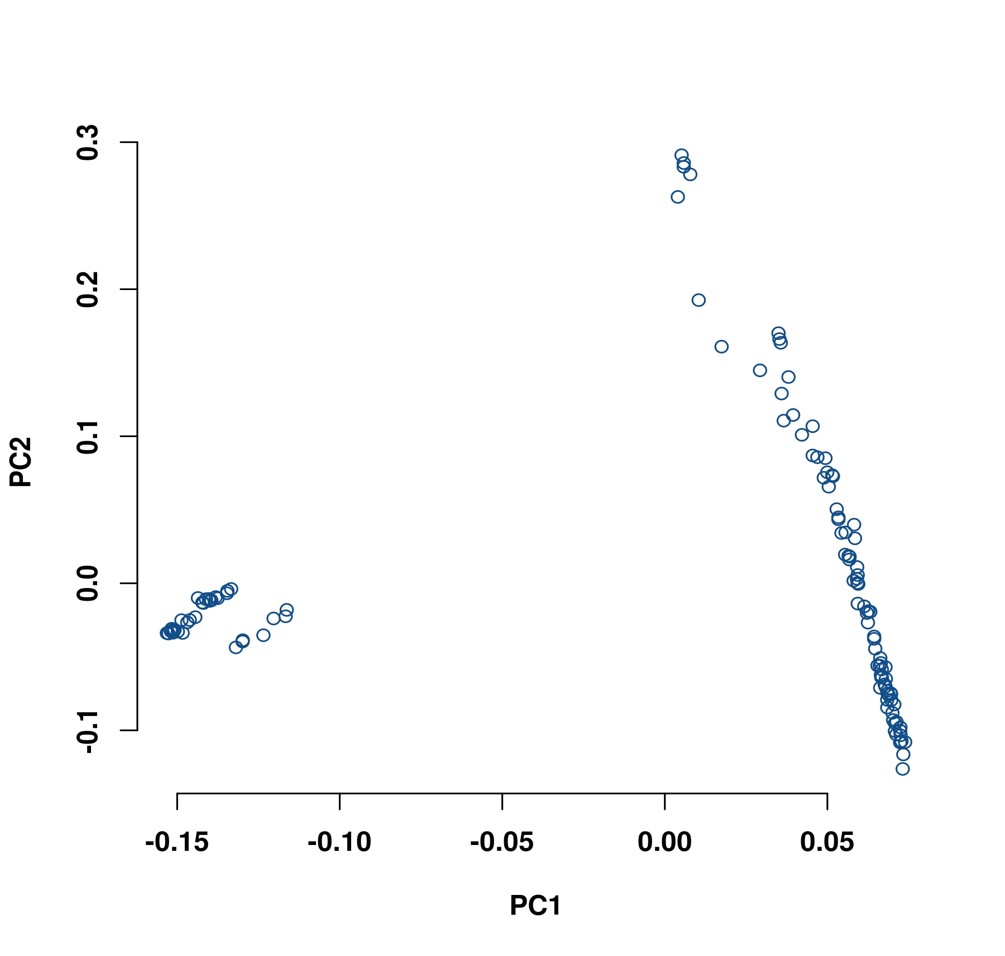

Supplement: Supplementary file 5 [file Image_5.jpeg]

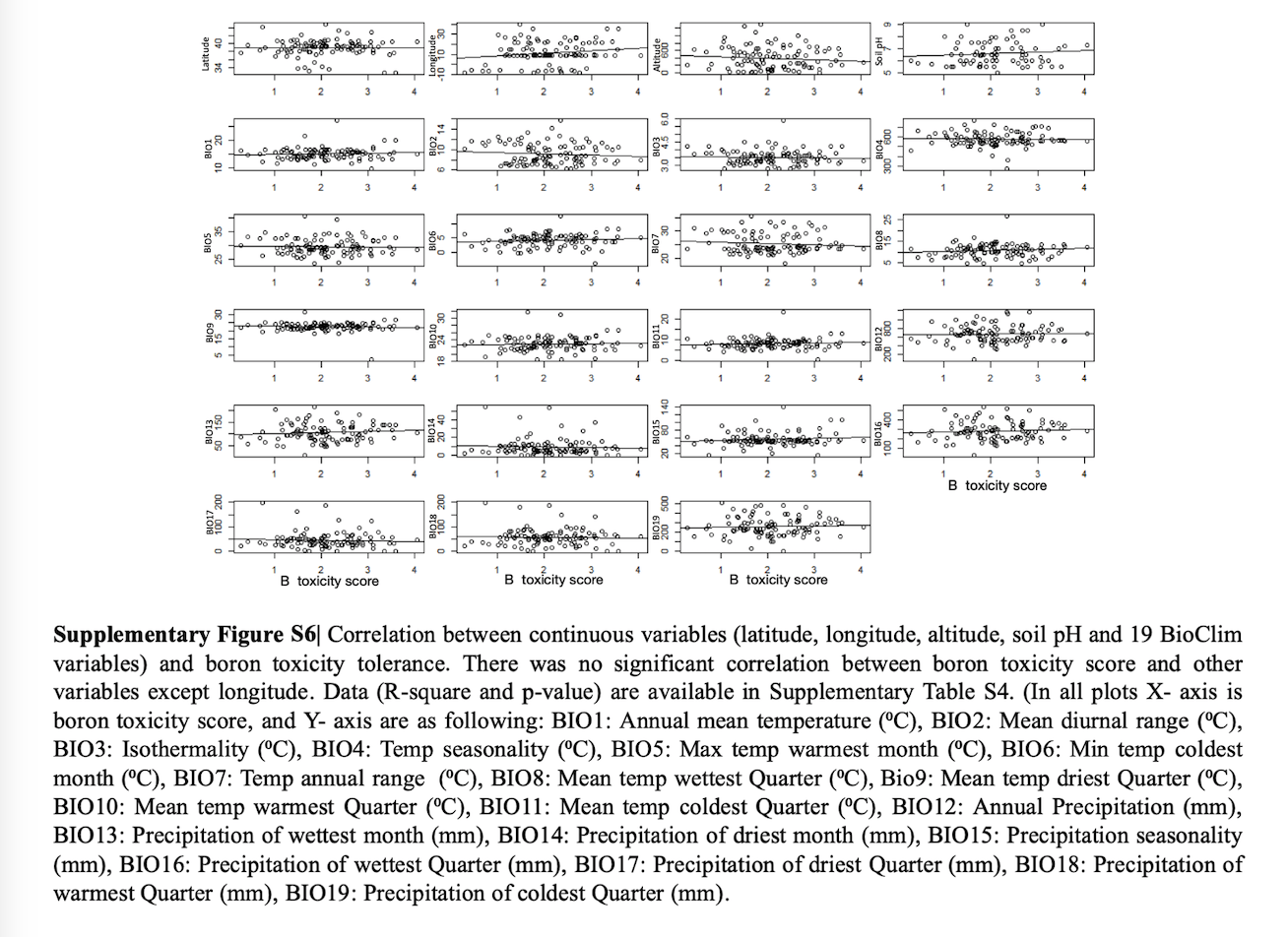

Supplement: Supplementary file 6 [file Image_6.png]

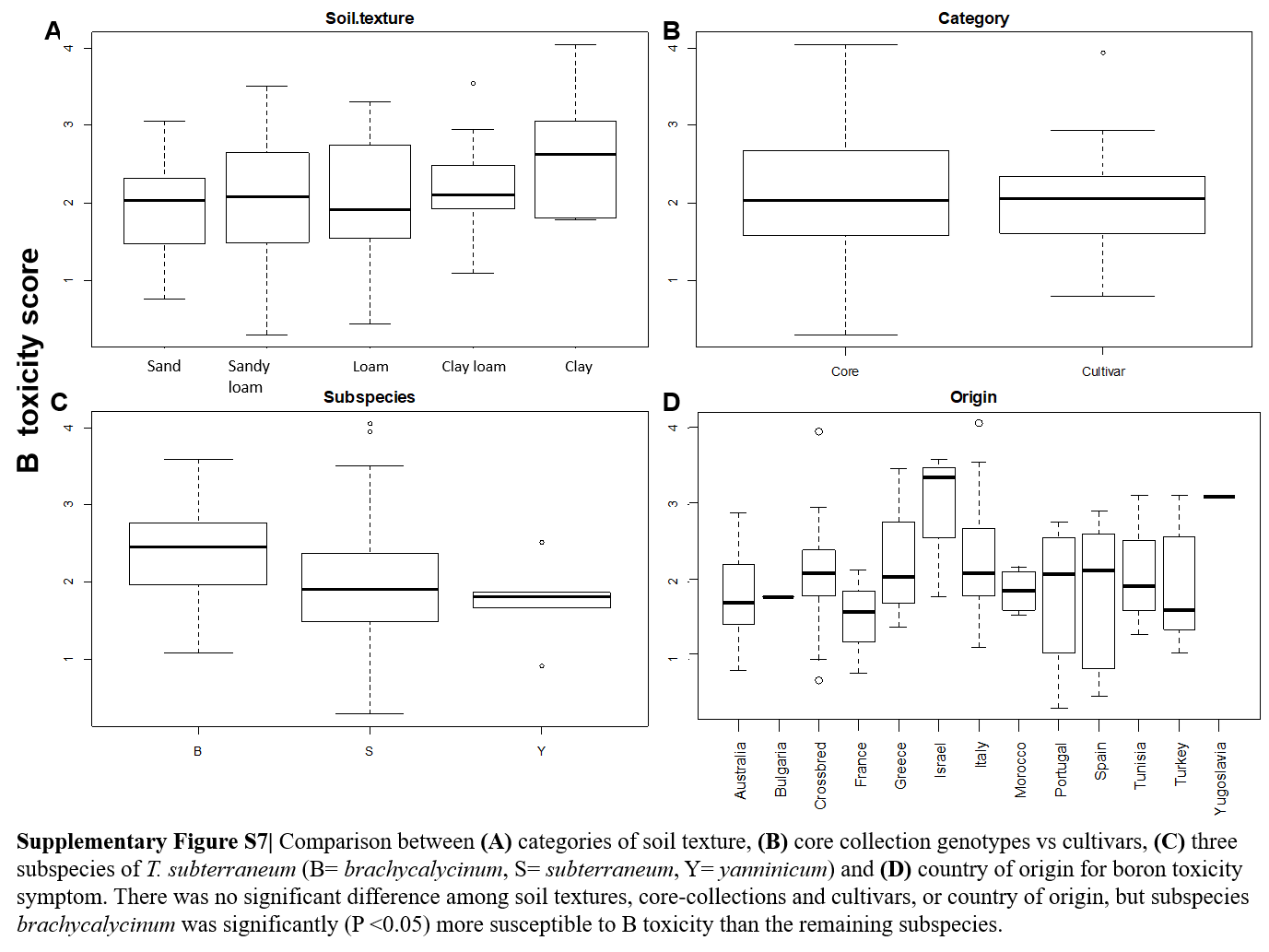

Supplement: Supplementary file 7 [file Image_7.tif]

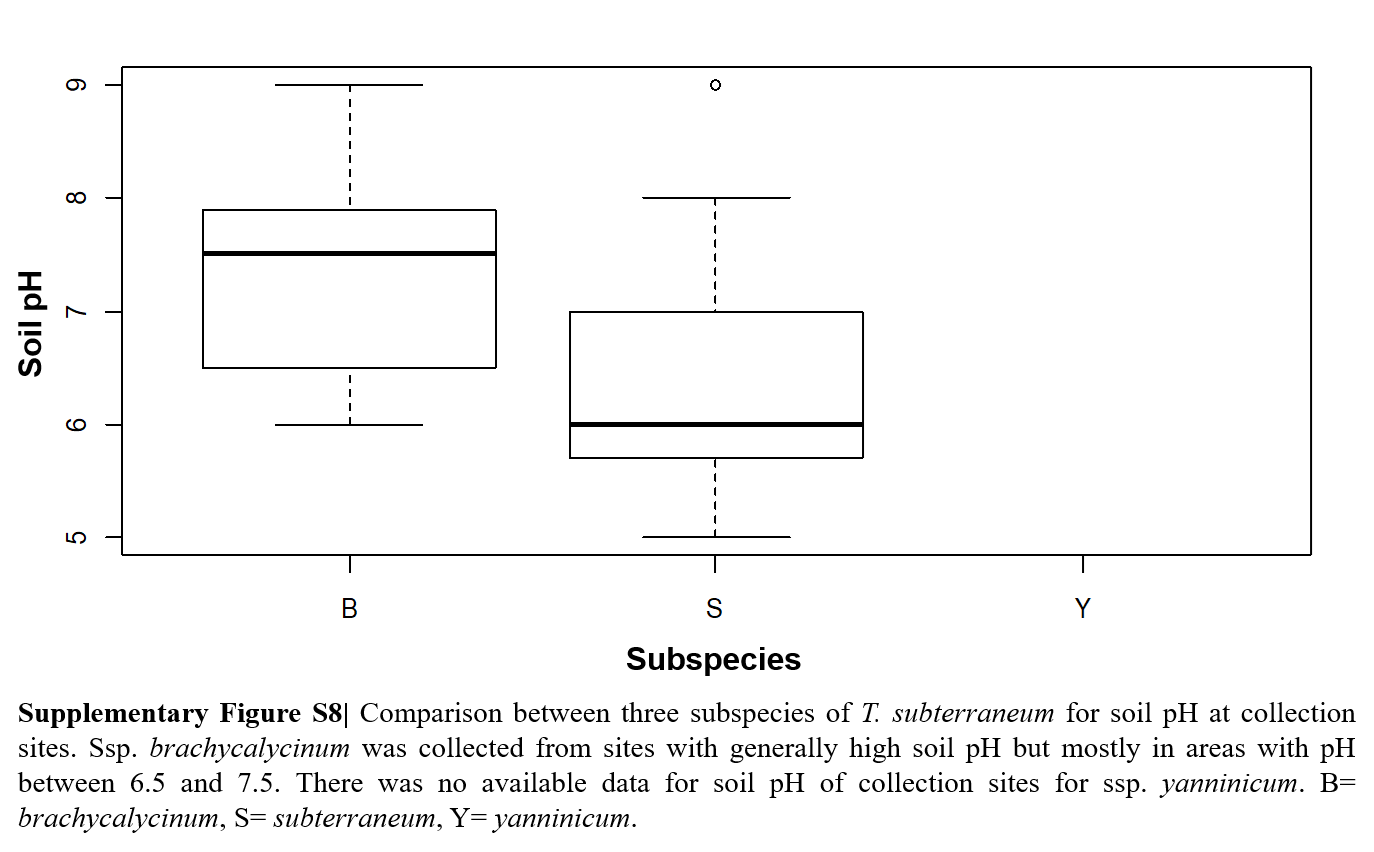

Supplement: Supplementary file 8 [file Image_8.tif]
